# Using molecular taxonomy to identify *Scinax* (Anura: Hylidae): New distribution records and implications for Neotropical biodiversity

**DOI:** 10.1590/1678-4685-GMB-2025-0053

**Published:** 2026-04-20

**Authors:** Lidia Nogueira, Reydson Reis, Paulo Roberto Antunes de Mello Affonso, Christine Strussmann, Iracilda Sampaio

**Affiliations:** 1 Instituto Federal de Educação, Ciência e Tecnologia da Bahia, Jequié, BA, Brazil.; 2 Universidade Federal do Pará, Programa de Pós-Graduação em Genética e Biologia Molecular, Belém, PA, Brazil.; 3 Instituto Federal de Educação, Ciência e Tecnologia do Pará, Belém, PA, Brazil.; 4 Universidade Estadual do Sudoeste da Bahia, Departamento de Ciências Biológicas, Jequié, BA, Brazil.; 5 Universidade Federal de Mato Grosso, Faculdade de Medicina Veterinária, Cuiabá, MT, Brazil.; 6 Universidade Federal do Pará, Instituto de Estudos Costeiros, Bragança, PA, Brazil.

**Keywords:** Conservation, genetic diversity, Neotropical amphibians, species delimitation

## Abstract

Traditional morphological identification is challenging in large and diverse groups, such as the genus *Scinax*, which comprises about 78 species, and molecular taxonomy emerges as an efficient tool for species delimitation. We generated 164 new mitochondrial 16S rRNA sequences from *Scinax* individuals collected in north and central-west Brazil, covering the Amazon biome, Amazon-Cerrado transition zones, and the Pantanal. After comparison with all sequences available in GenBank, 22 references representing the closest lineages were selected for detailed analyses. These were examined through phylogenetic inferences, genetic distances, and species delimitation methods (ASAP, ABGD, GMYC, bPTP). The analyses confirmed the genetic identity of individuals belonging to *Scinax acuminatus*, *S. boesemani*, *S. fuscomarginatus*, *S. fuscovarius*, *S. jolyi*, *S. madeirae*, *S. nasicus*, *S. nebulosus*, *S. proboscideus*, *S. similis*, *Scinax* sp. 1, sp. 2, sp. 5, sp. 7, sp. 22, and sp. 27. This study also expanded the distribution of eight species, including described and undescribed taxa (*S. jolyi*, *S. proboscideus*, *S. similis*, *Scinax* sp. 2, *Scinax* sp. 5, *Scinax* sp. 7, *Scinax* sp. 21 and *Scinax* sp. 27), highlighting the importance of molecular approaches for clarifying biogeographic patterns. Our results reinforce the effectiveness of molecular taxonomy for *Scinax* identification and contribute to refining genus diversity knowledge, reducing distributional gaps in Neotropical amphibians.

## Introduction

The Neotropical region harbors the highest diversity of amphibians on the planet, including many species that have yet to be described. Recent studies estimate that approximately 60% of future terrestrial vertebrate species discoveries will occur in tropical forests, with around 32.8% of these discoveries being amphibians (Moura and Jetz, 2021). In Brazil, which stands out as one of the countries with the greatest potential for new discoveries, it is estimated that most of the still undescribed vertebrate species may consist of amphibians, highlighting the urgency of methodologies that expedite the identification of biodiversity (Moura and Jetz, 2021).

Traditional identification based on morphology has limitations, especially in groups with high morphological similarity and the occurrence of cryptic species (Bickford et al., 2007). 

In this context, molecular techniques have proven to be effective tools for species delimitation, enabling the recognition of cryptic taxa and the taxonomic revision of widely distributed groups, particularly in anurans (Fouquet et al., 2007 a ; Vieites et al., 2009; Jansen et al., 2011; Vacher et al., 2020; Nogueira et al., 2022; Araujo-Vieira et al., 2023). Additionally, the combination of morphological and molecular analyses not only enhances taxonomic identification but also improves our understanding of the evolution and biogeography of these groups, providing crucial insights for conservation (Carné and Vieites, 2024).

Molecular taxonomy is a powerful tool that not only aids in species identification but also reveals cryptic lineages, assesses population genetic structure, and elucidates biogeographic patterns (e.g., Koroiva et al., 2020; Santana et al., 2024). Among the most widely used molecular markers for amphibians, the mitochondrial 16S rRNA gene stands out due to its broad application in comparative studies and the extensive number of sequences available in public databases, which facilitates consistent analyses and reliable identifications (Vences et al., 2005; Fouquet et al., 2007 a , b; Vieites et al., 2009; Jansen et al., 2011; Nogueira et al., 2022; Araujo-Vieira et al., 2023).

Despite these advances, a major challenge in biodiversity studies is the Wallacean shortfall, which refers to the lack of precise knowledge about the geographic distribution of species (Hortal et al., 2015). This gap is especially critical in megadiverse regions such as the Amazon and the Cerrado, where insufficient occurrence data hinder accurate biogeographic analyses and conservation planning. Addressing this shortfall requires expanding occurrence records and refining species delimitation, particularly in groups with complex taxonomy like *Scinax*.

The genus *Scinax* (Anura: Hylidae) currently comprises 78 recognized species, distributed from eastern and southern Mexico to Argentina and Uruguay, as well as on islands such as Trinidad, Tobago, and Saint Lucia (Frost, 2025). A recent comprehensive review redefined the genus, restricting *Scinax* to most species of the former *S. ruber* clade and organizing them into 13 species groups, while transferring other lineages to the genera *Ololygon* and *Julianus*. Importantly, this work also revealed 57 candidate species-an increase of 44.2% in recognized diversity within the tribe-highlighting the historical underestimation of species richness and the taxonomic complexity of the group (Araujo-Vieira et al., 2023).

Morphological identification within the genus *Scinax* remains challenging, as many species exhibit similar morphology, particularly those belonging to the same species group. This can lead to misidentifications and an underestimation of species diversity (Pombal-Jr et al., 1995; Araujo-Vieira et al., 2023).

Therefore, this study uses molecular taxonomy to identify *Scinax* specimens collected from northern and central-western Brazil, expanding knowledge of the genus diversity and distribution. More than a taxonomic effort, this approach provides new geographic records and refines species delimitation, contributing to reducing the Wallacean shortfall (Hortal et al., 2015) by clarifying distributional limits in a group with complex taxonomy and high ecological relevance in Neotropical ecosystems.

## Material and Methods

### Sample collection

**Figure 1 -:**
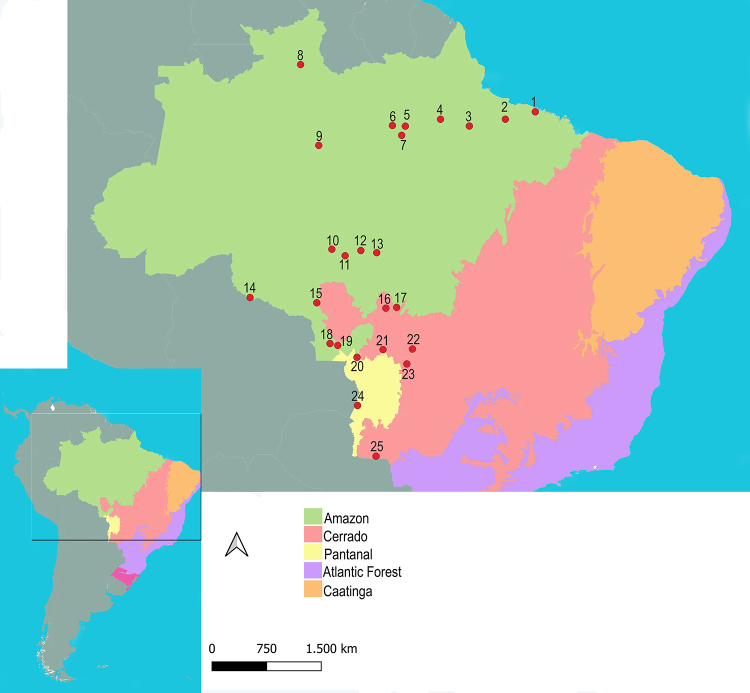
Map showing the sampling localities in the Amazon region, Pantanal, and transition areas with the Cerrado. Numbers correspond to the following municipalities: 1- Bragança (PA), 2- Barcarena (PA), 3- Portel (PA), 4- Almeirim (PA), 5- Alenquer (PA), 6- Óbidos (PA), 7- Alter do Chão (PA), 8- Manaus (AM), 9- Caracaraí (RR), 10- Colniza (MT), 11- Cotriguaçu (MT), 12- Apiacás (MT), 13- Paranaíta (MT), 14- Costa Marques (RO), 15- Vilhena (RO), 16- Lucas do Rio Verde (MT), 17- Nova Ubiratã (MT), 18- Pontes e Lacerda (MT), 19- Jauru (MT), 20- Cáceres (MT), 21- Cuiabá (MT), 22- Primavera do Leste (MT), 23- Rondonópolis (MT), 24- Corumbá (MS), 25- Bela Vista (MS).

We obtained 164 samples from different locations in the north and central-west regions of Brazil (Figure 1; Table S1). Due to the morphological similarity among congeners, not all specimens could be reliably identified at the species level during fieldwork. Therefore, species assignments were confirmed a posteriori through molecular analyses. For undescribed taxa (*Scinax* sp. 1, *Scinax* sp. 2, *Scinax* sp. 5, *Scinax* sp. 7, *Scinax* sp. 21, and *Scinax* sp. 27), we followed the nomenclature proposed by Araujo-Vieira et al. (2023).

For the molecular analyses, approximately 25 mg of muscle tissue was taken from the inner thigh of the individuals. The tissues were preserved in absolute ethanol (95%) and stored in a freezer at -20 ºC.

### DNA extraction and sequencing

DNA was extracted using the Wizard^®^ Genomic Purification kit (Promega), according to the manufacturer’s instructions. Amplification of the 16S mitochondrial gene was carried out by polymerase chain reaction (PCR), using primers 16S L1 (Palumbi, 1996) and 16S H1 (Varela et al., 2007). Each PCR reaction had a final volume of 25 μL, containing: 0.2 mM dNTPs, *Taq* polymerase buffer (1x), 2 mM MgCl₂, 200 ng of template DNA, 2 ng of each primer, 0.04 U/μL of Taq polymerase and 14.3 μL of ultrapure water.

The amplification conditions were as follows: initial denaturation at 95 ºC for 5 min, followed by 35 cycles of denaturation at 94 ºC for 40 s, annealing at 55 ºC for 40 s and extension at 72 ºC for 30 s, ending with a final extension at 72 ºC for 7 min. The quality of the PCR products was checked on a 1% agarose gel, stained with ethidium bromide.

Sequencing was carried out using the dideoxynucleotide termination method (Sanger et al., 1977), using reagents from the BigDye Terminator v3.1 Cycle Sequencing Kit (Applied Biosystems/Life Technologies). The sequencing reactions were analyzed on an ABI 3500 XL automatic sequencer (Life Technologies).

### Data analysis

We aligned the DNA sequences using the MAFFT v7 Q-INS-i algorithm (Katoh and Standley, 2013), implemented in the MEGA X software (Kumar et al., 2018). Regions of ambiguous alignment were excluded to avoid artificial noise in the dataset. To guide species selection for our analyses, we first compared our sequences with those analyzed by Araujo-Vieira et al. (2023), who carried out a taxonomic revision of the genus *Scinax* and constructed a phylogenetic tree including 77 species (38 not yet formally described). Based on this comparison, we selected 22 species that showed the greatest genetic similarity to our samples. For phylogenetic inference, the species *Ololygon* sp. was used as an outgroup.

The haplotypes were identified and compared using the DnaSP v6.12 program (Rozas et al., 2017), in order to remove identical sequences before the phylogenetic analyses. Only unique haplotypes were kept for the construction of the trees, ensuring the representativeness of the genetic diversity present in the samples.

Phylogenetic inference was conducted using Bayesian analysis in the MrBayes v3.1.3 software (Ronquist and Huelsenbeck, 2003). The best mutation model was estimated according to the Bayesian Information Criterion (BIC) in the software jModel Test 0.1 (Posada, 2008). Two runs (four chains each) with 10 million generations were performed with trees being sampled every 1000 generations. Adequate burn-in was determined by examining likelihood scores of the heated chains for convergence on stationarity (established in 25%), as well as the effective sample size values (>200) using Tracer v. 1.5 (Rambaut and Drummond, 2007). Strongly supported relationships were considered when posterior probability values were equal to or higher than 0.95. Additionally, a maximum likelihood (ML) tree was built using PhyML 3.0 (Guindon et al., 2010). For ML analyses, the best mutation model was estimated according to the Akaike Information Criterion (AIC) in the software jModel Test 0.1 (Posada, 2008). Non-parametric bootstrapping with heuristic searches of 1000 replicates was used to estimate the confidence values of branches in the ML tree.

Interspecific genetic distances were calculated using the Kimura-2-Parameters (K2P) model (Kimura, 1980), implemented in MEGA X (Kumar et al., 2018). The pairwise distance matrix is provided as Supplementary Material (Table S2). Based on this matrix, a heatmap of pairwise genetic distances was generated using Heatmapper (Babicki et al., 2016), employing average linkage clustering and Euclidean distance as the distance metric. Species delimitation was carried out using molecular diagnostic methods implemented in iTaxoTools 0.1 (Vences et al., 2021), including ASAP (Puillandre et al., 2021), ABGD (Puillandre *et al*., 2012), GMYC (Pons et al., 2006), and bPTP (Zhang et al., 2013).

## Results

A total of 164 sequences from 16 species of the genus *Scinax* were obtained in this study. The 16S mitochondrial gene fragment comprised 530 base pairs and revealed 219 variable sites. A summary of the sampled species, number of individuals (N), haplotypes (H), known distributions, and sampling localities is provided in Table 1. Molecular analyses confirmed the genetic identity of several *Scinax* species, recovering well-supported monophyletic groups for most taxa analyzed, including *S*. *nasicus*, *S*. *madeirae*, *S*. *fuscovarius*, *S*. *fuscomarginatus*, *S*. *boesemani*, *S*. *jolyi*, and *S*. *nebulosus*.

**Table 1 -:** Description of the collected species, number of individuals (N), number of haplotypes (H), species distribution according to the literature (Frost, 2025; Araújo-Vieira et al., 2023), and sampling locations in this study. Highlighted are the species whose distribution range has been expanded.

Specie	N	H	Species Distribution (Frost, 2025; Araújo-Vieira et al., 2023)	Sampling Locations
*S. acuminatus*	2	1	Southern Mato Grosso and Mato Grosso do Sul (Brazil), Paraguay, Bolivia (Santa Cruz), and northern Argentina	Cáceres - MT Corumbá - MS
*S. boesemani*	17	8	Northern Guyana, Suriname, and French Guiana, and Amazonas, Venezuela, to Acre, Amazonas, Pará, and Amapá, Brazil; expected in Amazonian Colombia, Peru, and Bolivia	Bragança - PA Barcarena - PA Alenquer - PA Óbidos - PA Manaus - AM
*S. fuscomarginatus*	12	3	Southern, central, and eastern Brazil, eastern Bolivia, eastern Paraguay, and northeastern Argentina	Lucas do Rio Verde - MT, Paranaíta - MT Bragança - PA Altamira - PA
*S. fuscovarius*	7	2	Southeastern Brazil from Alagoas south, northern Argentina, northern and eastern Paraguay, and Bolivia	Cáceres - MT Araputanga - MT Pontes e Lacerda - MT Jauru - MT Rondonópolis - MT
*S. jolyi*	15	6	Known from the type locality in northeastern French Guiana, Suriname, and Amapá, Brazil	Porto Velho - RO
*S. madeirae*	1	1	Rondônia, BR and eastern Bolivia	Vilhena - RO
*S. nasicus*	2	1	Paraguay, northern and central Argentina, northwestern to west central Uruguay, eastern Bolivia, and southern Brazil	Bela Vista - MS
*S. nebulosus*	12	4	Southeastern Venezuela, Guianas and the lower Amazon region to Alagoas in northeastern Brazil and through Mato Grosso to Amazonian Bolivia	Bragança - PA Caracaraí - RR PortoVelho - RO Portel - PA
*S. proboscideus*	2	1	Guyana, Suriname, and French Guiana; reported from Amapá, Brazil	Almeirim - PA
*S. similis*	14	6	Minas Gerais, Alagoas, Ceará, Mato Grosso, Tocantins, Maranhão, and Pará, reaching southern Suriname in the north, but also extending westwards to Bolivia, from where we included specimens from several localities in Santa Cruz and Beni, BOL	Cuiabá - MT Nova Ubiratã - MT Lucas do Rio Verde - MT Primavera do Leste - MT Corumbá - MS Altamira - PA
*Scinax* sp. 1	6	1	Amapá and Pará, Brazil, with localities along the rivers Tapajos, Xingu, and Amapari	Alter do Chão - PA
*Scinax* sp. 2	9	2	French Guiana	Óbidos - PA
*Scinax* sp. 5	6	1	From Piauí to Alagoas, northeastern Brazil	Barcarena - PA
*Scinax* sp. 7	2	1	Southeastern Peru	Porto Velho - RO
*Scinax* sp. 21	5	3	Serra do Navio, Amapá	Almeirim - PA
*Scinax* sp. 27	51	27	Pando and Santa Cruz (Bolivia), Amapá, Amazonas, Pará, Rondônia, Tocantins (Brazil), French Guiana, Loreto and Madre de Dios (Peru), Suriname, and Amazonas (Venezuela)	Porto Velho - RO Costa Marques - RO Cotriguaçu - MT Jauru - MT Araputanga - MT Colniza - MT Paranaíta -MT Apiacás - MT Manaus - AM Alter do Chão - PA Almerim - PA São Miguel - PA Altamira-PA Portel - PA Bragança - PA Alenquer - PA

**Figure 2 -:**
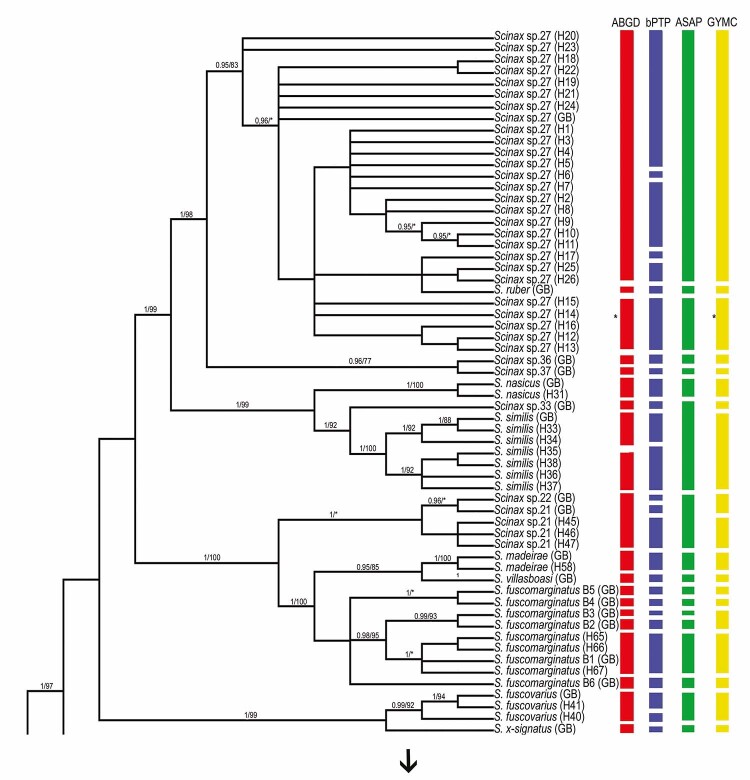
Bayesian inference (BI) topology with posterior probability values (pp > 95%) and maximum likelihood (ML) bootstrap support (>70%), based on the analysis of the mitochondrial 16S gene in *Scinax* species. Vertical bars correspond to each lineage considered a potential species. Species delimitation was inferred according to the ABGD, bPTP, ASAP, and GMYC methods.

**Figure 3 -:**
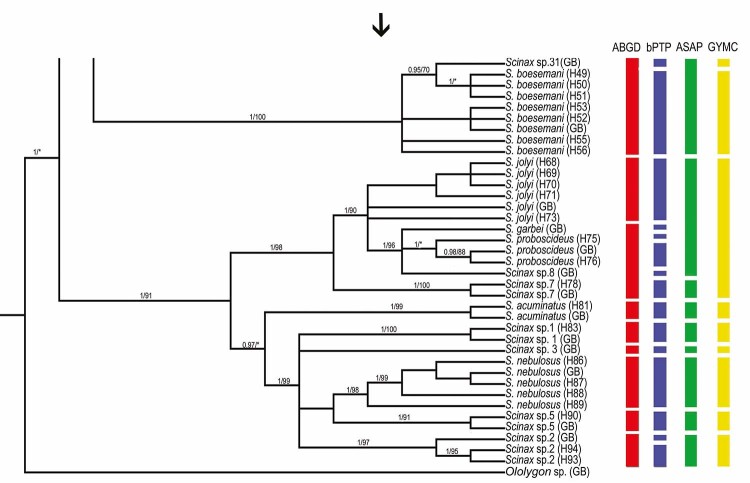
Continuation of the topology. Bayesian inference (BI) topology with posterior probability values (pp > 95%) and maximum likelihood (ML) bootstrap support (>70%), based on the analysis of the mitochondrial 16S gene in *Scinax* species. Vertical bars correspond to each lineage considered a potential species. Species delimitation was inferred according to the ABGD, bPTP, ASAP, and GMYC methods.

In contrast, cases of higher taxonomic complexity were observed in *Scinax* sp. 27, *S*. *similis*, and *Scinax* sp. 21, where different species delimitation methods showed disagreements regarding the number of lineages. These results highlight the effectiveness of molecular tools in validating species identities while also revealing potential intraspecific genetic structuring or species complexes within the genus *Scinax* (Figures 2 and 3).

**Figure 4 -:**
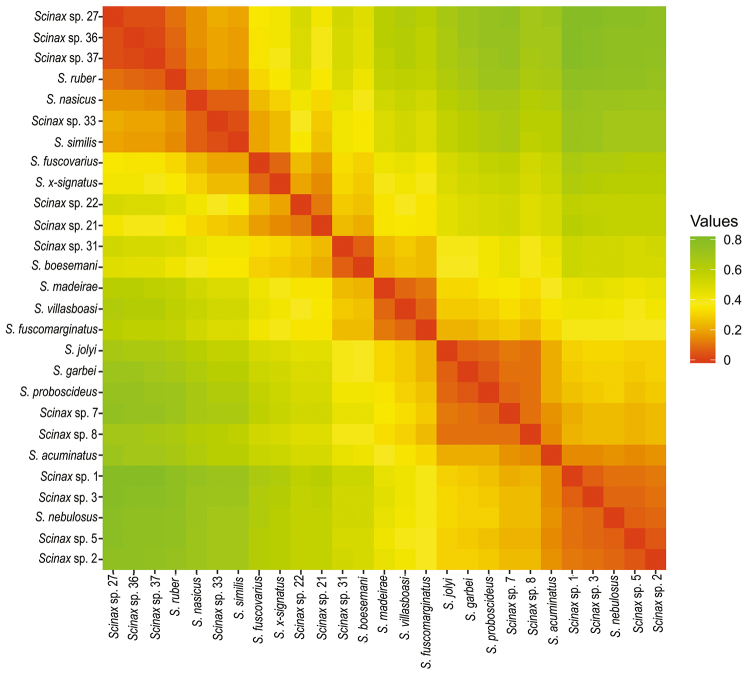
Heatmap of pairwise genetic distances (p-distances) among *Scinax* species based on the mitochondrial 16S gene, calculated in MEGA. Warmer colors (red-orange) indicate lower genetic distances (higher similarity), while cooler colors (yellow-green) indicate higher genetic distances (greater divergence). The color scale bar represents values ranging from 0 (no divergence) to 0.8 (maximum observed divergence).

The heatmap of pairwise genetic distances (Figure 4) revealed consistent patterns of divergence across taxa. Low divergence values (<0.05) were observed among closely related or potentially cryptic taxa, such as *Scinax* sp. 27, *S*. *ruber*, *Scinax* sp. 36, and *Scinax* sp. 37, reinforcing the complex structure of this lineage. Similarly, *S*. *similis* showed reduced divergence (<0.04) in relation to *Scinax* sp. 33, suggesting potential cases of recent diversification or species complexes. In contrast, higher distances (>0.15) were found between species belonging to different groups, such as *S*. *x-signatus* and *S*. *acuminatus*, confirming their deeper evolutionary divergence (Figure 4 and Table S2).

*Scinax* sp. 27 collected from different localities in the north and central-west regions of Brazil, in the Amazon biome and Amazon-Cerrado transition, presented 27 distinct haplotypes. The species delimitation analyses showed conflicting results. ABGD and GMYC grouped *Scinax* sp. 27, *S*. *ruber*, *Scinax* sp. 36, and *Scinax* sp. 37 into a single group. In contrast, bPTP identified five distinct groups within *Scinax* sp. 27, as well as recognizing *S*. *ruber*, *Scinax* sp. 36, and *Scinax* sp. 37 as separate units. ASAP indicated two distinct groupings within *Scinax* sp. 27: the first with samples from different localities in the north and central-west and the second composed of four haplotypes from Almeirim (PA, Amazon biome), Paranaíta (MT, Amazon-Cerrado transition), Apiacás (MT, Amazon-Cerrado transition) and Alter do Chão (PA, Amazon biome) (Figure 2). The genetic distances between *Scinax* sp. 27, *S*. *ruber*, *Scinax* sp. 36, and *Scinax* sp. 37 varied between 3% and 6% (Figure 4 and Table S2).

The sample of *S*. *nasicus* collected in Bonito (MS; Pantanal biome; H31) clustered with *S*. *nasicus* from GenBank with strong statistical support (1/100), and all the species delimitation analyses corroborated this relationship (Figure 2). 

The samples of *S*. *similis* were collected from various locations in the north and central-west of Brazil (Amazon biome, Amazon-Cerrado transition, and Pantanal). According to the ABGD and bPTP analyses, they comprise two clusters: one composed of two haplotypes from Cuiabá (MT, Amazon-Cerrado transition) and Corumbá (MS, Pantanal) and the other covering haplotypes from different localities in the north and central-west regions of Brazil, including Nova Ubiratã (MT), Lucas do Rio Verde (MT), Primavera do Leste (MT), Altamira (PA), and Vitória do Xingu (PA) (Amazon biome). However, the GMYC analysis combined all the samples into a single group, while the ASAP analysis included *Scinax* sp. 33 in this group (Figure 2). The genetic distance between *S*. *similis* and *Scinax* sp. 33 was 4% (Figure 4). 

Haplotypes H45, H46, and H47, from Almeirim (PA, Amazon biome), clustered with *Scinax* sp. 21 from GenBank with strong support. In the ABGD and ASAP analyses, these samples were also grouped with *Scinax* sp. 22, reflecting their low genetic divergence (3%). In contrast, bPTP separated *Scinax* sp. 21, *Scinax* sp. 22, and the new samples into distinct lineages, whereas GMYC merged all of them into a single cluster (Figures 2 and 4).

The sample of *S*. *madeirae* (H58), collected in Vilhena (Rondônia state - RO), showed high genetic similarity with a sample from GenBank, with strong statistical support (1/100), a relationship that was also confirmed in all the species delimitation analyses (Figure 2).

Samples of *S*. *fuscomarginatus*, collected in different locations in the north and central-west of Brazil, showed a strong genetic relationship with *S*. *fuscomarginatus* B1 from GenBank (Brusquetti et al., 2014), a result that was consistent in all the species delimitation analyses (Figure 2).

The individuals of *S*. *fuscovarius* from various localities in central-west of Brazil (Amazon biome and Amazon-Cerrado transition) formed a group with the GenBank samples of the same species, supported by both Bayesian and ML analyses (0.99/92), and confirmed by ABGD, ASAP, and GMYC (Figure 2).

The samples of *S*. *boesemani* collected in different locations in northern Brazil (Amazon biome) formed a single group, with strong statistical support (1/100). The ABGD and ASAP analyses indicated that *Scinax* sp. 31 belongs to this same group, with a genetic distance between the species of 4% (Figures 2 and 4).

The species *S*. *jolyi* (Amazon biome) was consistently recovered as a single cluster in the ABGD and bPTP analyses. The ASAP method, although recognizing *S*. *jolyi* as a distinct unit, indicated genetic proximity to *S*. *garbei*, *S*. *proboscideus*, and *Scinax* sp. 8, while GMYC additionally grouped *Scinax* sp. 7 within this cluster. Phylogenetic analyses (Figure 3) supported the close evolutionary relationship among these lineages, whereas species delimitation results demonstrated some disagreement regarding whether they represent independent species or a broader cluster. The pairwise genetic distances between these taxa ranged from 3% to 7% (Figures 3 and 4).

The bPTP analysis was the only one to distinguish *S*. *garbei* and *S*. *proboscideus* separately. For *S*. *proboscideus*, two subdivisions were evident: one containing a sample from GenBank and the other composed of a specimen from Alenquer - PA northwestern Pará State (Amazon biome) (Figure 3).

The genetic and species delimitation analyses (Figures 2 and 3) confirmed the identification of several taxa by their clustering with GenBank sequences. Haplotype H78, from Porto Velho (RO, Amazon biome), grouped with *Scinax* sp. 7 with strong support (1/99), while haplotype H81, from Cáceres (MT) and Corumbá (MS, Pantanal biome), clustered with *S*. *acuminatus*. Similarly, haplotype H83, from Alter do Chão (PA, Amazon biome), was identified as *Scinax* sp. 1. Samples of *S*. *nebulosus* from northern Brazil (Amazon biome) also clustered with GenBank sequences of the same species (1/98). In addition, haplotype H90, from Barcarena (PA, Amazon biome), was confirmed as *Scinax* sp. 5, and haplotypes H93 and H94, from Óbidos (PA, Amazon biome), grouped with *Scinax* sp. 2. All these relationships were recovered with high support in phylogenetic analyses and consistently corroborated by delimitation methods.

## Discussion

In this study, the analysis of 164 sequences representing 16 taxa of the genus *Scinax* provided a robust framework for evaluating species boundaries and biogeographic patterns within the genus. Phylogenetic inferences and species delimitation approaches were generally congruent in confirming the identity of most species, while also revealing cases of taxonomic complexity, particularly in lineages such as *S*. *similis*, *S*. *jolyi*, and *Scinax* sp. 27. Beyond clarifying these taxonomic issues, our results also contribute to refining the knowledge of the geographic ranges of several species, underscoring the importance of molecular data for addressing both systematic and biogeographic questions in Neotropical amphibians. These updated distribution ranges and sampling localities are summarized in Table 1.

*S*. *ruber*, a species widely distributed in northern Brazil, has been consistently identified as a complex of lineages (Fouquet et al., 2007 b ; Ferrão et al., 2016). Molecular and morphological evidence indicates that the taxon currently treated under this name encompasses at least six lineages, including *Scinax* sp. 27 (Araujo-Vieira et al., 2023). 

In our study, *Scinax* sp. 27 was recorded across a broad geographic range, with occurrences spanning the central, western, and eastern Amazon, as well as the southern Amazon and transitional areas with the Cerrado and Pantanal in Mato Grosso and Rondônia. This geographic breadth suggests that the lineage is more widespread and ecologically versatile than previously recognized, reinforcing the need for integrative approaches to clarify its taxonomic status and relationship with the broader *S*. *ruber* complex.

The species related to *S*. *nebulosus*, *Scinax* sp. 2 and *Scinax* sp. 5 also had their distribution expanded based on our results. *Scinax* sp. 2, previously recorded in French Guiana (Araujo-Vieira et al., 2023), was confirmed for the eastern Amazon (Óbidos, Pará), while *Scinax* sp. 5, known from northeastern Brazil (Piauí to Alagoas), was recorded for the first time in the eastern Amazon (Barcarena, Pará). These findings demonstrate how molecular data can uncover previously undetected distribution ranges, a pattern already reported for other Neotropical amphibians (Fouquet et al., 2007b; Ferrão et al., 2016).

Likewise, *Scinax* sp. 7, belonging to the *Scinax rostratus* group, had previous records in Peru and Bolivia, and our new samples from Porto Velho (Rondônia) expand its distribution into the southwestern Amazon of Brazil. Finally, *Scinax* sp. 21, known from Amapá, was also detected in Almeirim (Pará), extending its range within the eastern Amazon. 

Such records are not only relevant for species distribution updates, but also directly contribute to reducing the Wallacean shortfall (Hortal et al., 2015), by filling critical knowledge gaps on where species occur in poorly sampled Amazonian and transitional landscapes. These extensions reinforce the role of molecular approaches in revealing hidden diversity and refining the biogeographic patterns of Neotropical frogs, particularly in transitional zones of high environmental heterogeneity (Penhacek et al., 2024). Moreover, predictive tools such as species distribution models (SDMs) could further enhance these efforts by estimating potential ranges based on occurrence records and environmental variables, supporting both biogeographic inferences and conservation planning.

*S*. *similis* is widely distributed, with records across multiple Brazilian states (Minas Gerais, Alagoas, Ceará, Mato Grosso, Tocantins, Maranhão, and Pará) and extending into Suriname and Bolivia (Araujo-Vieira et al., 2023; Lopes et al., 2023; Frost, 2025). Our study not only confirms its occurrence in Pará and Mato Grosso but also provides the first record in the Pantanal biome (Corumbá, MS), thereby expanding its range to yet another major Neotropical biome. This finding reinforces the ecological breadth of the species, which is now documented across the Atlantic Forest, Caatinga, Cerrado, Amazonia, and Pantanal. 

At the same time, the species delimitation analyses revealed incongruent results: ABGD and bPTP recovered two clusters, GMYC grouped all samples as a single lineage, and ASAP additionally included *Scinax* sp. 33. Such discrepancies indicate genetic structuring within *S*. *similis* and support the hypothesis that it may comprise a species complex, emphasizing the importance of integrative taxonomic studies to clarify its evolutionary relationships.

*S*. *proboscideus*, a member of the *S. rostratus* group, was previously recorded in the interior of the Guianas (Guyana, Suriname, and French Guiana) and in northern Brazil, including Amapá (Frost, 2025). Our data add a new record from Óbidos, in the eastern Amazon (Pará), which refines the known distribution of the species within Brazil and highlights the importance of filling sampling gaps in the region. Such records are particularly valuable in the Amazon, where incomplete sampling often obscures real biogeographic patterns (Fouquet et al., 2007 b ; Ferrão et al., 2016).

The different species delimitation methods (ABGD, ASAP, bPTP, and GMYC) yielded incongruent results, with GMYC merging lineages, ABGD and ASAP being more conservative, and bPTP identifying additional subdivisions. These inconsistencies, which are expected given the distinct assumptions of each method (Puillandre et al., 2012; Zhang et al., 2013), were especially evident for lineages such as *Scinax* sp. 27, *S. similis*, Scinax sp. 21, and *S. jolyi*. 

Such patterns likely reflect both the complex evolutionary history of these taxa and methodological limitations. Although the mitochondrial 16S marker has proven useful for species identification and comparison with GenBank data, it has limited resolution for more complex taxonomic scenarios (Chan et al., 2022). In these cases, additional data will be required. Future studies should incorporate complementary mitochondrial (e.g., COI, cyt-b) and nuclear markers, as well as larger sample sizes per taxon, to provide a more integrative and reliable framework for species delimitation. Such approaches will not only refine the taxonomy of these groups but also help uncover potential cryptic diversity within the genus.

The use of molecular tools allows comparison with reference databases, facilitating accurate species identification. This approach is especially valuable in biodiversity hotspots, where many species may not be recognized due to taxonomic complexity. In addition, the construction and continuous expansion of genetic sequence databases is fundamental for future studies, allowing for more comprehensive and refined comparisons (Hebert et al., 2003; Nogueira et al., 2016; Nogueira *et al*., 2022; Santana et al., 2024). In the Amazon, recent assessments have shown that traditional morphological approaches are often insufficient to capture the true extent of amphibian diversity, reinforcing the importance of molecular tools in revealing hidden lineages and informing conservation strategies (Penhacek et al., 2024). 

Therefore, our study reinforces the usefulness of molecular tools in identifying and delimiting species of the genus *Scinax*, expands the known distribution of several taxa, and contributes to the genetic databases available, providing a valuable basis for future taxonomic and ecological research in Neotropical amphibians.

## Supplementary material

The following online material is available for this article:

Table S1 - Description of the samples: species, code, geographic coordinates, haplotype, GenBank accession number and reference.

Table S2 - Interspecific nucleotide divergence in the 16S gene among *Scinax* species based on the K2P model.

## Data Availability

All DNA sequences generated in this study have been deposited in GenBank under the accession numbers provided in the manuscript (Table S1).
