# Supplementary material for: Using molecular taxonomy to identify Scinax (Anura: Hylidae): New distribution records and implications for Neotropical biodiversity
Source: Genet Mol Biol. 2026 Apr 20;49(1):e20250053. doi: 10.1590/1678-4685-GMB-2025-0053 (PMC13131051; doi:10.1590/1678-4685-GMB-2025-0053)
Supplement: Table S1 - [file 1415-4757-GMB-49-01-e20250053-s1.pdf]

Genetics and Molecular Biology
https://doi.org/10.1590/1678-4685-GMB-2025-0053

Supplementary Material to "Using molecular taxonomy to identify *Scinax* (Anura: Hylidae): New distribution records and implications for Neotropical biodiversity"

Table S1 - Description of the samples: species, code, geographic coordinates, haplotype, GenBank accession number and reference.

Specie	Code	Sampled Location	Geographic Coordinates	Haplotype	GenBank	Reference
Ololygon sp.	495	Ilhéus, BA, BR	14°47′50″S, 39°2′ 8″W		OK161110	Nogueira *et al.*, 2022
S. acuminatus	367	Caceres, MT, BR	16° 4′ 1″ S, 57° 41′ 12″ W	H81	PX396985	This study
S. acuminatus	342	Corumbá, MS, BR	19° 0′ 35″ S, 57° 39′ 17″ W	H81	PX396985	This study
S. acuminatus	MACN38649	Corrientes, ARG	27°28′S, 58°50′W	H82	OQ934395	Araujo-Vieira *et al.*, 2023
S. boesemani	376	Barcarena, PA, BR	1° 31′ 8″ S, 48° 37′ 1″ W	H49	PX396823	This study
S. boesemani	173	Barcarena, PA, BR	1° 3′ 57″ S, 46° 47′ 22″ W	H50	PX396824	This study
S. boesemani	399	Barcarena, PA, BR	1° 31′ 8″ S, 48° 37′ 1″ W	H51	PX396825	This study
S. boesemani	472	Alenquer, PA, BR	1° 56′ 33″ S, 54° 44′ 15″ W	H52	PX396826	This study
S. boesemani	467	Alenquer, PA, BR	1° 56′ 33″ S, 54° 44′ 15″ W	H52	PX396827	This study
S. boesemani	468	Alenquer, PA, BR	1° 56′ 33″ S, 54° 44′ 15″ W	H52	PX396828	This study
S. boesemani	473	Alenquer, PA, BR	1° 56′ 33″ S, 54° 44′ 15″ W	H52	PX396829	This study
S. boesemani	474	Alenquer, PA, BR	1° 56′ 33″ S, 54° 44′ 15″ W	H53	PX396830	This study
S. boesemani	454	Óbidos, PA, BR	1° 54′ 7″ S, 55° 31′ 11″ W	H54	PX396831	This study
S. boesemani	113	Manaus, AM, BR	3° 6′ 26″ S, 60° 1′ 34″ W	H55	PX396832	This study
S. boesemani	112	Manaus, AM, BR	3° 6′ 26″ S, 60° 1′ 34″ W	H55	PX396833	This study
S. boesemani	114	Manaus, AM, BR	3° 6′ 26″ S, 60° 1′ 34″ W	H55	PX396834	This study
S. boesemani	476	Alenquer, PA, BR	1° 56′ 33″ S, 54° 44′ 15″ W	H56	PX396835	This study
S. boesemani	475	Alenquer, PA, BR	1° 56′ 33″ S, 54° 44′ 15″ W	H56	PX396836	This study
S. boesemani	471	Alenquer, PA, BR	1° 56′ 33″ S, 54° 44′ 15″ W	H56	PX396837	This study
S. boesemani	470	Alenquer, PA, BR	1° 56′ 33″ S, 54° 44′ 15″ W	H56	PX396838	This study
S. boesemani	418	Bragança, PA, BR	1° 3′ 57″ S, 46° 47′ 22″ W	H49	PX396839	This study

Specie	Code	Sampled Location	Geographic Coordinates	Haplotype	GenBank	Reference
S. boesemani	A164194	Potaro-Siparuni, GUY	4°42′N, 59°24′W	H54	OQ934424	Araujo-Vieira *et al.*, 2023
Ololygon sp.	495	Ilhéus, BA, BR	14°47′50″S, 39°2′ 8″W		OK161110	Nogueira *et al.*, 2022
S. acuminatus	367	Caceres, MT, BR	16° 4′ 1″ S, 57° 41′ 12″ W	H81	PX396985	This study
S. acuminatus	342	Corumbá, MS, BR	19° 0′ 35″ S, 57° 39′ 17″ W	H81	PX396985	This study
S. acuminatus	MACN38649	Corrientes, ARG	27°28′S, 58°50′W	H82	OQ934395	Araujo-Vieira *et al.*, 2023
S. boesemani	376	Barcarena, PA, BR	1° 31′ 8″ S, 48° 37′ 1″ W	H49	PX396823	This study
S. boesemani	173	Barcarena, PA, BR	1° 3′ 57″ S, 46° 47′ 22″ W	H50	PX396824	This study
S. boesemani	399	Barcarena, PA, BR	1° 31′ 8″ S, 48° 37′ 1″ W	H51	PX396825	This study
S. boesemani	472	Alenquer, PA, BR	1° 56′ 33″ S, 54° 44′ 15″ W	H52	PX396826	This study
S. boesemani	467	Alenquer, PA, BR	1° 56′ 33″ S, 54° 44′ 15″ W	H52	PX396827	This study
S. boesemani	468	Alenquer, PA, BR	1° 56′ 33″ S, 54° 44′ 15″ W	H52	PX396828	This study
S. boesemani	473	Alenquer, PA, BR	1° 56′ 33″ S, 54° 44′ 15″ W	H52	PX396829	This study
S. boesemani	474	Alenquer, PA, BR	1° 56′ 33″ S, 54° 44′ 15″ W	H53	PX396830	This study
S. boesemani	454	Óbidos, PA, BR	1° 54′ 7″ S, 55° 31′ 11″ W	H54	PX396831	This study
S. boesemani	113	Manaus, AM, BR	3° 6′ 26″ S, 60° 1′ 34″ W	H55	PX396832	This study
S. boesemani	112	Manaus, AM, BR	3° 6′ 26″ S, 60° 1′ 34″ W	H55	PX396833	This study
S. boesemani	114	Manaus, AM, BR	3° 6′ 26″ S, 60° 1′ 34″ W	H55	PX396834	This study
S. boesemani	476	Alenquer, PA, BR	1° 56′ 33″ S, 54° 44′ 15″ W	H56	PX396835	This study
S. boesemani	475	Alenquer, PA, BR	1° 56′ 33″ S, 54° 44′ 15″ W	H56	PX396836	This study
S. boesemani	471	Alenquer, PA, BR	1° 56′ 33″ S, 54° 44′ 15″ W	H56	PX396837	This study
S. boesemani	470	Alenquer, PA, BR	1° 56′ 33″ S, 54° 44′ 15″ W	H56	PX396838	This study
S. boesemani	418	Bragança, PA, BR	1° 3′ 57″ S, 46° 47′ 22″ W	H49	PX396839	This study
S. boesemani	A164194	Potaro-Siparuni, GUY	4°42′N, 59°24′W	H54	OQ934424	Araujo-Vieira *et al.*, 2023

Specie	Code	Sampled Location	Geographic Coordinates	Haplotype	GenBank	Reference
S. fuscomarginatus	409	Lucas do Rio Verde, MT, BR	13° 3′ 48″ S, 55° 55′ 16″ W	H65	PX396840	This study
S. fuscomarginatus	362	Lucas do Rio Verde, MT, BR	13° 3′ 48″ S, 55° 55′ 16″ W	H65	PX396841	This study
S. fuscomarginatus	391	Paranaíta, MT, BR	9° 40′ 23″ S, 56° 28′ 50″ W	H66	PX396842	This study
S. fuscomarginatus	174	Bragança, PA, BR	1° 3′ 57″ S, 46° 47′ 22″ W	H67	PX396843	This study
S. fuscomarginatus	543	Altamira, PA, BR	3° 11′ 41″ S, 52° 12′ 33″ W	H67	PX396844	This study
S. fuscomarginatus	540	Altamira, PA, BR	3° 11′ 41″ S, 52° 12′ 33″ W	H67	PX396845	This study
S. fuscomarginatus	537	Altamira, PA, BR	3° 11′ 41″ S, 52° 12′ 33″ W	H67	PX396846	This study
S. fuscomarginatus	553	Altamira, PA, BR	3° 11′ 41″ S, 52° 12′ 33″ W	H67	PX396847	This study
S. fuscomarginatus	361	Lucas do Rio Verde, MT, BR	13° 3′ 48″ S, 55° 55′ 16″ W	H67	PX396848	This study
S. fuscomarginatus	552	Altamira, PA, BR	3° 11′ 41″ S, 52° 12′ 33″ W	H67	PX396849	This study
S. fuscomarginatus	547	Altamira, PA, BR	3° 11′ 41″ S, 52° 12′ 33″ W	H67	PX396850	This study
S. fuscomarginatus	541	Altamira, PA, BR	3° 11′ 41″ S, 52° 12′ 33″ W	H67	PX396851	This study
S. fuscomarginatus B1	CAB5751	Santa Elena, VEM	4°36′N, 61°6′W	H67	KJ004120	Brusquetti *et al.*, 2014
S. fuscomarginatus B2	CFBH23345	Caetite, BA, BR	14°4′S, 42°28′W	H63	KJ004131	Brusquetti *et al.*, 2014
S. fuscomarginatus B3	CHUNB38023	Paranã, TO, BR	12°36′S, 47°52′W	H62	KJ004122	Brusquetti *et al.*, 2014
S. fuscomarginatus B4	MNKA9624	Caparu, BOL	17°48′00″S, 63°10′00″W	H61	KJ004114	Brusquetti *et al.*, 2014
S. fuscomarginatus B5	CFBH10049	São Paulo, SP, BR	23°33′S, 46°38′W	H60	KJ004110	Brusquetti *et al.*, 2014
S. fuscomarginatus B6	CTMZ6684	Paranaita, MT, BR	9°39′S, 56°28′W	H62	KJ004173	Brusquetti *et al.*, 2014
S. fuscovarius	349	Araputanga, MT, BR	15° 28′ 3″ S, 58° 21′ 22″ W	H40	PX396852	This study
S. fuscovarius	358	Caceres, MT, BR	16° 4′ 1″ S, 57° 41′ 12″ W	H40	PX396853	This study
S. fuscovarius	363	Caceres, MT, BR	16° 4′ 1″ S, 57° 41′ 12″ W	H40	PX396854	This study
S. fuscovarius	370	Pontes e Lacerda, MT, BR	15° 14′ 3″ S, 59° 19′ 52″ W	H40	PX396855	This study
S. fuscovarius	411	Jauru, MT, BR	15° 20′ 21″ S, 58° 51′ 45″ W	H40	PX396856	This study

Specie	Code	Sampled Location	Geographic Coordinates	Haplotype		Reference
S. fuscovarius	335	Araputanga, MT, BR	15° 28′ 3″ S, 58° 21′ 22″ W	H40	PX396857	This study
S. fuscovarius	403	Rondonopolis, MT, BR	16° 28′ 17″ S, 54° 38′ 14″ W	H41	PX396858	This study
S. fuscovarius	CFBHT00795	Sao Joao D'Alianca, GO, BR	14°42′S, 47°31′W	H39	KU495550	Lyra *et al.*, 2016
S. garbei	SCF1144	Ucayali, PE	8°22′S, 74°34′W	H74	OQ934572	Araujo-Vieira *et al.*, 2023
S. jolyi	58	PortoVelho, RO, BR	8° 45′ 43″ S, 63° 54′ 7″W	H68	PX396859	This study
S. jolyi	46	PortoVelho, RO, BR	8° 45′ 43″ S, 63° 54′ 7″W	H68	PX396860	This study
S. jolyi	90	PortoVelho, RO, BR	8° 45′ 43″ S, 63° 54′ 7″W	H68	PX396861	This study
S. jolyi	102	PortoVelho, RO, BR	8° 45′ 43″ S, 63° 54′ 7″W	H68	PX396862	This study
S. jolyi	49	PortoVelho, RO, BR	8° 45′ 43″ S, 63° 54′ 7″W	H69	PX396863	This study
S. jolyi	38	PortoVelho, RO, BR	8° 45′ 43″ S, 63° 54′ 7″W	H70	PX396864	This study
S. jolyi	80	PortoVelho, RO, BR	8° 45′ 43″ S, 63° 54′ 7″W	H71	PX396865	This study
S. jolyi	426	PortoVelho, RO, BR	8° 45′ 43″ S, 63° 54′ 7″W	H73	PX396866	This study
S. jolyi	73	PortoVelho, RO, BR	8° 45′ 43″ S, 63° 54′ 7″W	H73	PX396867	This study
S. jolyi	71	PortoVelho, RO, BR	8° 45′ 43″ S, 63° 54′ 7″W	H73	PX396868	This study
S. jolyi	55	PortoVelho, RO, BR	8° 45′ 43″ S, 63° 54′ 7″W	H73	PX396869	This study
S. jolyi	425	PortoVelho, RO, BR	8° 45′ 43″ S, 63° 54′ 7″W	H73	PX396870	This study
S. jolyi	72	PortoVelho, RO, BR	8° 45′ 43″ S, 63° 54′ 7″W	H73	PX396871	This study
S. jolyi	52	PortoVelho, RO, BR	8° 45′ 43″ S, 63° 54′ 7″W	H73	PX396872	This study
S. jolyi	444	Almeirim, PA, BR	1° 31′ 14″ S, 52° 34′ 53″ W	H75	PX396873	This study
S. jolyi	PG136	French Guiana, GUF	4°0′N, 53°0′W	H72	OQ934617	Araujo-Vieira *et al.*, 2023
S. madeirae	366	Vilhena, RO, BR	12° 44′ 3″ S, 60° 8′ 41″ W	H58	PX396874	This study
S. madeirae	AS0034	Tucumancito, BOL	16°25′00″S, 62°03′00″W	H57	JF789947	
S. nasicus	394	Bela Vista, MS, BR	22° 4′ 57″ S, 56° 31′ 33″ W	H31	PX396875	This study
S. nasicus	359	Bela Vista, MS, BR	22° 4′ 57″ S, 56° 31′ 33″ W	H31	PX396876	This study
S. nasicus	CFBH14245	Bonito, MS, BR	21°7′S, 56°28′W	H31		Araujo-Vieira *et al.*, 2023
S. nebulosus	172	Bragança, PA, BR	1° 3′ 57″ S, 46° 47′ 22″ W	H86	PX396877	This study
S. nebulosus	169	Bragança, PA, BR	1° 3′ 57″ S, 46° 47′ 22″ W	H86	PX396878	This study

Specie	Code	Sampled Location	Geographic Coordinates	Haplotype		Reference
S. nebulosus	170	Bragança, PA, BR	1° 3′ 57″ S, 46° 47′ 22″ W	H86	PX396879	This study
S. nebulosus	16	Caracaraí, RR, BR	1° 49′ 13″ N, 61° 7′ 57″ W	H86	PX396880	This study
S. nebulosus	33	Caracaraí, RR, BR	1° 49′ 13″ N, 61° 7′ 57″ W	H86	PX396881	This study
S. nebulosus	171	Bragança, PA, BR	1° 3′ 57″ S, 46° 47′ 22″ W	H86	PX396882	This study
S. nebulosus	167	Bragança, PA, BR	1° 3′ 57″ S, 46° 47′ 22″ W	H86	PX396883	This study
S. nebulosus	166	Bragança, PA, BR	1° 3′ 57″ S, 46° 47′ 22″ W	H86	PX396884	This study
S. nebulosus	64	PortoVelho, RO, BR	8° 45′ 43″ S, 63° 54′ 7″W	H88	PX396885	This study
S. nebulosus	168	Bragança, PA, BR	1° 3′ 57″ S, 46° 47′ 22″ W	H87	PX396886	This study
S. nebulosus	479	Portel, PA, BR	1° 56′ 32″ S, 50° 48′ 33″ W	H89	PX396887	This study
S. nebulosus	478	Portel, PA, BR	1° 56′ 32″ S, 50° 48′ 33″ W	H89	PX396888	This study
S. nebulosus	PS337	Ananindeua, PA, BR	1°22′S, 48°22′W	H87	OQ934641	Araujo-Vieira *et al.*, 2023
S. proboscideus	447	Almeirim, PA, BR	1° 31′ 14″ S, 52° 34′ 53″ W	H76	PX396889	This study
S. proboscideus	450	Almeirim, PA, BR	1° 31′ 14″ S, 52° 34′ 53″ W	H76	PX396890	This study
S. proboscideus	465	Alenquer, PA, BR	1° 56′ 33″ S, 54° 44′ 15″ W	H76	PX396891	This study
S. proboscideus	USNM531493	Potaro-Siparuni, GUY	4°42′N, 59°24′W	H77	OQ934661	Araujo-Vieira *et al.*, 2023
S. ruber	IWK113	Potaro-Siparuni, GUY	4°42′N, 59°24′W	H30	OQ934678	Araujo-Vieira *et al.*, 2023
S. similis	398	Cuiabá, MT, BR	15° 35′ 56″ S, 56° 5′ 42″ W	H33	PX396892	This study
S. similis	317	Corumbá, MS, BR	19° 0′ 35″ S, 57° 39′ 17″ W	H34	PX396893	This study
S. similis	414	Nova Ubiratã, MT, BR	13° 0′ 53″ S, 55° 15′ 50″ W	H35	PX396894	This study
S. similis	387	Lucas do Rio Verde, MT, BR	13° 3′ 48″ S, 55° 55′ 16″ W	H36	PX396895	This study
S. similis	546	Altamira, PA, BR	3° 11′ 41″ S, 52° 12′ 33″ W	H37	PX396896	This study
S. similis	352	Lucas do Rio Verde, MT, BR	13° 3′ 48″ S, 55° 55′ 16″ W	H38	PX396897	This study
S. similis	375	Lucas do Rio Verde, MT, BR	13° 3′ 48″ S, 55° 55′ 16″ W	H38	PX396898	This study
S. similis	539	Altamira, PA, BR	3° 11′ 41″ S, 52° 12′ 33″ W	H37	PX396899	This study
S. similis	482	Vitória do Xingu, PA, BR	2° 53′ 2″ S, 52° 0′ 17″ W	H37	PX396900	This study
S. similis	549	Altamira, PA, BR	3° 11′ 41″ S, 52° 12′ 33″ W	H37	PX396901	This study

Specie	Code	Sampled Location	Geographic Coordinates	Haplotype		Reference
S. similis	365	Primavera do Leste, MT, BR	15° 33′ 35″ S, 54° 17′ 50″ W	H37	PX396902	This study
S. similis	415	Primavera do Leste, MT, BR	15° 33′ 35″ S, 54° 17′ 50″ W	H38	PX396903	This study
S. similis	542	Altamira, PA, BR	3° 11′ 41″ S, 52° 12′ 33″ W	H37	PX396904	This study
S. similis	371	Primavera do Leste, MT, BR	15° 33′ 35″ S, 54° 17′ 50″ W	H37	PX396905	This study
S. similis	CFBH14386	Chapada dos Guimarães, MT, BR	15°27′S, 55°45′W	H33	OQ934701	Araujo-Vieira *et al.*, 2023
Scinax sp. 1	109	Alter do Chão, PA, BR	2° 30′ 15″ S, 35° 04′ 15″ W	H83	PX396906	This study
Scinax sp. 1	108	Alter do Chão, PA, BR	2° 30′ 15″ S, 35° 04′ 15″ W	H83	PX396907	This study
Scinax sp. 1	107	Alter do Chão, PA, BR	2° 30′ 15″ S, 35° 04′ 15″ W	H83	PX396908	This study
Scinax sp. 1	111	Alter do Chão, PA, BR	2° 30′ 15″ S, 35° 04′ 15″ W	H83	PX396909	This study
Scinax sp. 1	110	Alter do Chão, PA, BR	2° 30′ 15″ S, 35° 04′ 15″ W	H83	PX396910	This study
Scinax sp. 1	106	Alter do Chão, PA, BR	2° 30′ 15″ S, 35° 04′ 15″ W	H83	PX396911	This study
Scinax sp. 1	BM079	Vitória do Xingu, PA, BR	2° 53′ 2″ S, 52° 0′ 17″ W	H84	KDQF01001822	Vacher et al. 2020
Scinax sp.2	460	Óbidos, PA, BR	1° 54′ 7″ S, 55° 31′ 11″ W	H93	PX396912	This study
Scinax sp.2	464	Óbidos, PA, BR	1° 54′ 7″ S, 55° 31′ 11″ W	H93	PX396913	This study
Scinax sp.2	463	Óbidos, PA, BR	1° 54′ 7″ S, 55° 31′ 11″ W	H93	PX396914	This study
Scinax sp.2	459	Óbidos, PA, BR	1° 54′ 7″ S, 55° 31′ 11″ W	H93	PX396915	This study
Scinax sp.2	461	Óbidos, PA, BR	1° 54′ 7″ S, 55° 31′ 11″ W	H93	PX396916	This study
Scinax sp.2	457	Óbidos, PA, BR	1° 54′ 7″ S, 55° 31′ 11″ W	H93	PX396917	This study
Scinax sp.2	458	Óbidos, PA, BR	1° 54′ 7″ S, 55° 31′ 11″ W	H93	PX396918	This study
Scinax sp.2	462	Óbidos, PA, BR	1° 54′ 7″ S, 55° 31′ 11″ W	H94	PX396919	This study
Scinax sp.2	456	Óbidos, PA, BR	1° 54′ 7″ S, 55° 31′ 11″ W	H93	PX396920	This study
Scinax sp. 2	AMNH-A141126	Upper Demerara-Berbice, GUY	5°0′N, 58°15′W	H92	OQ934751	Araujo-Vieira *et al.*, 2023
Scinax sp. 3	AB418	Corumbá, MS, BR	19° 0′ 35″ S, 57° 39′ 17″ W	H85	OQ934752	Araujo-Vieira *et al.*, 2023
Scinax sp.5	380	Barcarena, PA, BR	1° 31′ 8″ S, 48° 37′ 1″ W	H90	PX396921	This study

Specie	Code	Sampled Location	Geographic Coordinates	Haplotype		Reference
Scinax sp.5	377	Barcarena, PA, BR	1° 31′ 8″ S, 48° 37′ 1″ W	H90	PX396922	This study
Scinax sp.5	379	Barcarena, PA, BR	1° 31′ 8″ S, 48° 37′ 1″ W	H90	PX396923	This study
Scinax sp.5	382	Barcarena, PA, BR	1° 31′ 8″ S, 48° 37′ 1″ W	H90	PX396924	This study
Scinax sp.5	381	Barcarena, PA, BR	1° 31′ 8″ S, 48° 37′ 1″ W	H90	PX396925	This study
Scinax sp.5	378	Barcarena, PA, BR	1° 31′ 8″ S, 48° 37′ 1″ W	H90	PX396926	This study
Scinax sp. 5	CFBH15626	Acailândia, MA, BR	4°57′S, 47°30′W	H91	OQ934754	Araujo-Vieira *et al.*, 2023
Scinax sp.07	429	PortoVelho, RO, BR	8° 45′ 43″ S, 63° 54′ 7″W	H78	PX396927	This study
Scinax sp.07	428	PortoVelho, RO, BR	8° 45′ 43″ S, 63° 54′ 7″W	H78	PX396928	This study
Scinax sp. 7	WED57696	Madre de Dios, PE	12°36′S, 69°11′W	H80	OQ934758	Araujo-Vieira *et al.*, 2023
Scinax sp. 8	MTR968395	Apiacás, MT, BR	9° 32′ 40″ S, 57° 27′ 4″ W	H79	OQ934759	Araujo-Vieira *et al.*, 2023
Scinax sp. 21	448	Almeirim, PA, BR	1° 31′ 14″ S, 52° 34′ 53″ W	H45	PX396929	This study
Scinax sp. 21	446	Almeirim, PA, BR	1° 31′ 14″ S, 52° 34′ 53″ W	H45	PX396930	This study
Scinax sp. 21	451	Almeirim, PA, BR	1° 31′ 14″ S, 52° 34′ 53″ W	H46	PX396931	This study
Scinax sp. 21	477	Almeirim, PA, BR	1° 31′ 14″ S, 52° 34′ 53″ W	H47	PX396932	This study
Scinax sp. 21	445	Almeirim, PA, BR	1° 31′ 14″ S, 52° 34′ 53″ W	H47	PX396933	This study
Scinax sp. 21	MTR13734	Serra do Navio, AP, BR	0°53′N, 52°0′W	H44	KDQF010032 46	Vacher *et al.*, 2020
Scinax sp. 22	I4	St Laurent, GUF	5°30′N, 54°2′W	H43	EF217494	Fouquet *et al.*, 2007
Scinax sp. 27	97	PortoVelho, RO, BR	8° 45′ 43″ S, 63° 54′ 7″W	H1	PX396934	This study
Scinax sp. 27	84	PortoVelho, RO, BR	8° 45′ 43″ S, 63° 54′ 7″W	H1	PX396935	This study
Scinax sp. 27	41	PortoVelho, RO, BR	8° 45′ 43″ S, 63° 54′ 7″W	H1	PX396936	This study
Scinax sp. 27	69	PortoVelho, RO, BR	8° 45′ 43″ S, 63° 54′ 7″W	H1	PX396937	This study
Scinax sp. 27	83	PortoVelho, RO, BR	8° 45′ 43″ S, 63° 54′ 7″W	H1	PX396938	This study
Scinax sp. 27	61	PortoVelho, RO, BR	8° 45′ 43″ S, 63° 54′ 7″W	H1	PX396939	This study
Scinax sp. 27	67	PortoVelho, RO, BR	8° 45′ 43″ S, 63° 54′ 7″W	H1	PX396940	This study
Scinax sp. 27	93	PortoVelho, RO, BR	8° 45′ 43″ S, 63° 54′ 7″W	H1	PX396941	This study
Scinax sp. 27	65	PortoVelho, RO, BR	8° 45′ 43″ S, 63° 54′ 7″W	H1	PX396942	This study
Scinax sp. 27	86	PortoVelho, RO, BR	8° 45′ 43″ S, 63° 54′ 7″W	H1	PX396943	This study
Scinax sp. 27	60	PortoVelho, RO, BR	8° 45′ 43″ S, 63° 54′ 7″W	H1	PX396944	This study
Scinax sp. 27	75	PortoVelho, RO, BR	8° 45′ 43″ S, 63° 54′ 7″W	H1	PX396945	This study

Specie	Code	Sampled Location	Geographic Coordinates	Haplotype		Reference
Scinax sp. 27	74	PortoVelho, RO, BR	8° 45′ 43″ S, 63° 54′ 7″W	H1	PX396946	This study
Scinax sp. 27	59	PortoVelho, RO, BR	8° 45′ 43″ S, 63° 54′ 7″W	H1	PX396947	This study
Scinax sp. 27	360	Cotriguaçu, MT, BR	9° 51′ 30″ S, 58° 24′ 45″ W	H2	PX396948	This study
Scinax sp. 27	424	PortoVelho, RO, BR	8° 45′ 43″ S, 63° 54′ 7″W	H3	PX396949	This study
Scinax sp. 27	37	Manaus, AM, BR	3° 6′ 26″ S, 60° 1′ 34″ W	H4	PX396950	This study
Scinax sp. 27	81	PortoVelho, RO, BR	8° 45′ 43″ S, 63° 54′ 7″W	H4	PX396951	This study
Scinax sp. 27	76	PortoVelho, RO, BR	8° 45′ 43″ S, 63° 54′ 7″W	H4	PX396952	This study
Scinax sp. 27	66	PortoVelho, RO, BR	8° 45′ 43″ S, 63° 54′ 7″W	H5	PX396953	This study
Scinax sp. 27	85	PortoVelho, RO, BR	8° 45′ 43″ S, 63° 54′ 7″W	H6	PX396954	This study
Scinax sp. 27	42	PortoVelho, RO, BR	8° 45′ 43″ S, 63° 54′ 7″W	H6	PX396955	This study
Scinax sp. 27	384	Jauru, MT, BR	15° 20′ 21″ S, 58° 51′ 45″ W	H7	PX396956	This study
Scinax sp. 27	368	Araputanga, MT, BR	15° 28′ 3″ S, 58° 21′ 22″ W	H7	PX396957	This study
Scinax sp. 27	423	Costa Marques, RO, BR	12° 24′ 56″ S, 64° 13′ 17″ W	H8	PX396958	This study
Scinax sp. 27	416	Colniza, MT, BR	9° 24′ 1″ S, 59° 1′ 57″ W	H9	PX396959	This study
Scinax sp. 27	385	Cotriguaçu, MT, BR	9° 51′ 30″ S, 58° 24′ 45″ W	H10	PX396960	This study
Scinax sp. 27	330	Colniza, MT, BR	9° 24′ 1″ S, 59° 1′ 57″ W	H11	PX396961	This study
Scinax sp. 27	440	Alter do Chão, PA, BR	2° 30′ 15″ S, 35° 04′ 15″ W	H12	PX396962	This study
Scinax sp. 27	439	Alter do Chão, PA, BR	2° 30′ 15″ S, 35° 04′ 15″ W	H13	PX396963	This study
Scinax sp. 27	453	Almeirim, PA, BR	1° 31′ 14″ S, 52° 34′ 53″ W	H14	PX396964	This study
Scinax sp. 27	452	Almeirim, PA, BR	1° 31′ 14″ S, 52° 34′ 53″ W	H15	PX396965	This study
Scinax sp. 27	396	Paranaíta, MT, BR	9° 40′ 23″ S, 56° 28′ 50″ W	H16	PX396966	This study
Scinax sp. 27	347	Apiacas, MT, BR	9° 32′ 40″ S, 57° 27′ 4″ W	H16	PX396967	This study
Scinax sp. 27	421	Manaus, AM, BR	3° 6′ 26″ S, 60° 1′ 34″ W	H17	PX396968	This study
Scinax sp. 27	419	Manaus, AM, BR	3° 6′ 26″ S, 60° 1′ 34″ W	H17	PX396969	This study
Scinax sp. 27	234	São Miguel, PA, BR	1° 37′ 40″ S, 47° 28′ 55″ W	H18	PX396970	This study
Scinax sp. 27	481	Portel, PA, BR	1° 56′ 32″ S, 50° 48′ 33″ W	H19	PX396971	This study
Scinax sp. 27	545	Altamira, PA, BR	3° 11′ 41″ S, 52° 12′ 33″ W	H20	PX396972	This study
Scinax sp. 27	544	Altamira, PA, BR	3° 11′ 41″ S, 52° 12′ 33″ W	H20	PX396973	This study
Scinax sp. 27	555	Altamira, PA, BR	3° 11′ 41″ S, 52° 12′ 33″ W	H21	PX396974	This study

Specie	Code	Sampled Location	Geographic Coordinates	Haplotype		Reference
Scinax sp. 27	235	São Miguel, PA, BR	1° 37′ 40″ S, 47° 28′ 55″ W	H22	PX396975	This study
Scinax sp. 27	538	Altamira, PA, BR	3° 11′ 41″ S, 52° 12′ 33″ W	H23	PX396976	This study
Scinax sp. 27	556	Altamira, PA, BR	3° 11′ 41″ S, 52° 12′ 33″ W	H24	PX396977	This study
Scinax sp. 27	550	Altamira, PA, BR	3° 11′ 41″ S, 52° 12′ 33″ W	H23	PX396978	This study
Scinax sp. 27	239	Bragança, PA, BR	1° 3′ 57″ S, 46° 47′ 22″ W	H24	PX396979	This study
Scinax sp. 27	233	São Miguel, PA, BR	1° 37′ 40″ S, 47° 28′ 55″ W	H24	PX396980	This study
Scinax sp. 27	266	São Miguel, PA, BR	1° 37′ 40″ S, 47° 28′ 55″ W	H24	PX396981	This study
Scinax sp. 27	466	Alenquer, PA, BR	1° 56′ 33″ S, 54° 44′ 15″ W	H25	PX396982	This study
Scinax sp. 27	449	Almeirim, PA, BR	1° 31′ 14″ S, 52° 34′ 53″ W	H26	PX396983	This study
Scinax sp. 27	420	Manaus, AM, BR	3° 6′ 26″ S, 60° 1′ 34″ W	H27	PX396984	This study
Scinax sp. 27	MHNLS21081	Alto Orinico, VEM	3°49′N, 65°41′W	H27		Araujo-Vieira *et al.*, 2023
Scinax sp. 33	A141125	Dubulay Ranch on the Berbice River, GUY	5°00′N, 59°00′W.	H32	OQ934843	Araujo-Vieira *et al.*, 2023
Scinax sp. 36	NMP70895	Beni, BOL	14°30′S, 66°15′W	H28	OQ934849	Araujo-Vieira *et al.*, 2023
Scinax sp. 37	QCAZ51062	Orellana, ECU	0°28′S, 76°59′W	H29	MH662477	Araujo-Vieira *et al.*, 2023
S. villasboasi	CHUNB 40156	Serra do Cachimbo, PA, BR	8°45′S, 55°0′W	H59	KJ004108	Araujo-Vieira *et al.*, 2023
S. x-signatus	UESC11046	Paulo Afonso, BA, BR	9°24′39″S, 38°14′9″W	H42	OK161174	Nogueira *et al.*, 2022

References

Araujo-Vieira K, Lourenço ACC, Lacerda JVA, Lyra ML, Blotto BL, Ron SR, Baldo D, Pereyra OM, Suárez-Mayorga AM, Baêta D *et al.* (2023) Treefrog diversity in the Neotropics: Phylogenetic relationships of *Scinaxini* (Anura: Hylidae: Hylinae). South Am J Herp 27:1-143.

Brusquetti F, Jansen M, Barrio-Amorós C, Segalla MV and Haddad CFB (2014) Taxonomic review of *Scinax fuscomarginatus* (Lutz, 1925) and related species (Anura: Hylidae). Zool J Linn Soc 171:783-821.

Fouquet A, Vences M, Salducci M, Meyer A, Marty C, Blanc M and Gilles A (2007b) Revealing cryptic diversity using molecular phylogenetics and phylogeography in frogs of the *Scinax ruber* and *Rhinella margaritifera* species groups. Mol Phylogenet Evol 43:567-582.

Lyra ML, Haddad CF and de Azeredo-Espin AML (2017) Meeting the challenge of DNA barcoding Neotropical amphibians: Polymerase chain reaction optimization and new COI primers. Mol Ecol Resour 17:966-980.

Nogueira L, Rodrigues Filho LFDS, Solé M, Affonso PRADM, Siqueira S and Sampaio I (2022) DNA barcode reveals candidate species of *Scinax* and *Ololygon* (Anura: Hylidae) in Atlantic Forest. Genet Mol Biol 45:e20210177.

Vacher JP, Chave J, Ficetola FG, Sommeria-Klein G, Tao S, Thébaud C, Blanc M, Camacho A, Cassimiro J, Colston TJ *et al.* (2020) Large-scale DNA-based survey of frogs in Amazonia suggests a vast underestimation of species richness and endemism. J Biogeogr 47:1781-1791.
