# Supplementary material for: Using molecular taxonomy to identify Scinax (Anura: Hylidae): New distribution records and implications for Neotropical biodiversity
Source: Genet Mol Biol. 2026 Apr 20;49(1):e20250053. doi: 10.1590/1678-4685-GMB-2025-0053 (PMC13131051; doi:10.1590/1678-4685-GMB-2025-0053)
Supplement: Table S2 - [file 1415-4757-GMB-49-01-e20250053-s2.pdf]

Genetics and Molecular Biology
https://doi.org/10.1590/1678-4685-GMB-2025-0053

Supplementary Material to “Using molecular taxonomy to identify *Scinax* (Anura: Hylidae): New distribution records and implications for Neotropical biodiversity”

Table S2 – Interspecific nucleotide divergence in the 16S gene among *Scinax* species based on the K2P model.

	Taxon	1	2	3	4	5	6	7	8	9	10	11	12	13	14	15	16	17	18	19	20	21	22	23	24	25	26
1	*Scinax* sp.27																										
2	*Scinax* sp.36	0,05																									
3	*Scinax* sp.37	0,04	0,03																								
4	*S. ruber*	0,05	0,07	0,06																							
5	*S. nasicus*	0,08	0,08	0,08	0,12																						
6	*Scinax* sp.33	0,10	0,10	0,09	0,14	0,07																					
7	*S. similis*	0,09	0,09	0,09	0,13	0,05	0,04																				
8	*S. fuscovarius*	0,13	0,15	0,14	0,18	0,13	0,13	0,13																			
9	*S. x-signatus*	0,16	0,16	0,15	0,22	0,15	0,14	0,14	0,07																		
10	*Scinax* sp.22	0,17	0,19	0,19	0,19	0,17	0,18	0,16	0,16	0,18																	
11	*Scinax* sp.21	0,13	0,15	0,15	0,18	0,13	0,14	0,12	0,12	0,14	0,03																
12	*Scinax* sp.31	0,16	0,15	0,16	0,22	0,14	0,15	0,15	0,12	0,14	0,23	0,16															
13	*S. boesemani*	0,14	0,15	0,15	0,18	0,13	0,13	0,13	0,12	0,13	0,20	0,15	0,04														
14	*S. madeirae*	0,18	0,18	0,17	0,22	0,17	0,16	0,16	0,15	0,18	0,21	0,14	0,17	0,18													
15	*S. villasboasi*	0,18	0,18	0,19	0,23	0,18	0,16	0,16	0,15	0,17	0,22	0,15	0,19	0,18	0,08												
16	*S. fuscomarginatus*	0,17	0,18	0,17	0,22	0,17	0,16	0,16	0,15	0,16	0,21	0,14	0,16	0,16	0,09	0,08											
17	*S. jolyi*	0,17	0,16	0,15	0,21	0,17	0,17	0,17	0,15	0,16	0,21	0,16	0,17	0,16	0,18	0,19	0,19										
18	*S. garbei*	0,18	0,17	0,17	0,22	0,17	0,17	0,17	0,16	0,17	0,25	0,19	0,17	0,16	0,19	0,18	0,18	0,04									
19	*S. proboscideus*	0,19	0,18	0,18	0,23	0,18	0,19	0,19	0,17	0,18	0,24	0,19	0,17	0,17	0,19	0,21	0,19	0,03	0,03								
20	*Scinax* sp.7	0,18	0,17	0,17	0,23	0,18	0,19	0,18	0,16	0,17	0,23	0,17	0,20	0,18	0,21	0,21	0,20	0,05	0,07	0,06							
21	*Scinax* sp.8	0,17	0,17	0,16	0,21	0,17	0,18	0,17	0,15	0,16	0,22	0,17	0,17	0,16	0,18	0,19	0,19	0,04	0,04	0,04	0,07						
22	*S. acuminatus*	0,16	0,17	0,16	0,20	0,16	0,16	0,16	0,15	0,17	0,21	0,15	0,17	0,17	0,18	0,20	0,17	0,10	0,10	0,10	0,10	0,10					
23	*Scinax* sp.1	0,20	0,19	0,19	0,24	0,17	0,18	0,17	0,18	0,18	0,22	0,17	0,21	0,20	0,20	0,21	0,21	0,11	0,12	0,14	0,12	0,12	0,10				
24	*Scinax* sp.3	0,20	0,20	0,20	0,25	0,17	0,18	0,17	0,17	0,19	0,20	0,16	0,19	0,18	0,19	0,21	0,20	0,12	0,13	0,15	0,12	0,13	0,09	0,05			
25	*S. nebulosus*	0,18	0,18	0,18	0,23	0,17	0,18	0,17	0,17	0,18	0,20	0,16	0,21	0,19	0,18	0,19	0,19	0,12	0,13	0,14	0,11	0,13	0,11	0,07	0,07		
26	*Scinax* sp.5	0,19	0,19	0,19	0,24	0,17	0,18	0,17	0,16	0,17	0,22	0,16	0,20	0,19	0,19	0,19	0,19	0,11	0,12	0,13	0,11	0,12	0,09	0,05	0,06	0,05	
27	*Scinax* sp.2	0,18	0,18	0,17	0,24	0,16	0,18	0,17	0,17	0,16	0,20	0,16	0,20	0,19	0,18	0,19	0,20	0,11	0,12	0,13	0,12	0,13	0,10	0,05	0,06	0,06	0,04
